# Genome-wide identification, characterization and expression of HSP 20 gene family in dove

**DOI:** 10.3389/fgene.2022.1011676

**Published:** 2022-10-04

**Authors:** Zhe Hou, Ang Li, Changbing Huang

**Affiliations:** ^1^ College of Landscape Engineering, SuZhou Polytechnic Institute of Agriculture, Suzhou, China; ^2^ Key Laboratory of Southwest China Wildlife Resources Conservation (Ministry of Education), College of Life Science, China West Normal University, Nanchong, China

**Keywords:** Davidia involucrata, heat shock protein 20, heat stress, gene family, defense mechanisms

## Abstract

*Davidia involucrata* is a significant living fossil with high abiotic stress tolerance. Although heat shock protein 20 (HSP20) has already been linked to heat stress, nothing is known about HSP20 family protein activities in *D. involucrata*. The functional dynamics of the *D. involucrata* HSP20 (*DiHSP20*) gene family were identified and characterized using a thorough genome-wide investigation. From the genome of *D. involucrata*, a total of 42 HSP20 genes were identified, which are distributed across 16 chromosomes. The *DiHSP20* proteins were grouped into seven separate subfamilies by our phylogenetic analysis, which was validated by the conserved motif composition and gene structure studies. Segmental duplication events were shown to play a crucial role in the expansion of the *DiHSP20* gene family. Synteny analysis revealed that 19 *DiHSP20* genes of *D. involucrata* shared a syntenic connection with *Arabidopsis* genes, 39 with *C. acuminata* genes, and just 6 with *O. sativa* genes. Additionally, heat stress differently enhanced the expression levels of *D. involucrata* HSP20 genes. After 1 hour of heat treatment, the expression levels of most *DiHSP20* genes, particularly *DiHSP20-7*, *DiHSP20-29*, *DiHSP20-30*, *DiHSP20-32*, and *DiHSP20-34*, were dramatically increased, suggestted that they might be employed as heat tolerance candidate genes. Overall, these findings add to our knowledge of the HSP20 family genes and provide helpful information for breeding heat stress resistance in *D. involucrata*.

## Introduction

Plants are often influenced by a wide range of abiotic and biotic stresses during growth, including cold, drought, extreme heat, high salinity, and infestation by pests and pathogens (Hu et al., 2021). Over the long period of evolution, plants have evolved their unique defense mechanisms to avoid exposure to unfavorable environments, such as different physiological, molecular, morphological, and adaptive mechanisms (Wang et al., 2004). Heat shock proteins (HSP) are widely found in prokaryotic and eukaryotic organisms as a molecular chaperone and are involved in many developmental processes and responses to environmental stress. They are induced to be expressed or increased when the organism is exposed to environmental stresses, such as infection by pathogens, heat stress, reactive oxygen species (ROS), drought, and heavy metals (Kandoth et al., 2011). In general, HSP and denatured proteins produced under stress conditions maintain or restore intracellular homeostasis through folding, assembly, translocation, and degradation, thereby reducing the damage caused by stress to the organism (Maimbo et al., 2007; De Souza Resende et al., 2022).

In recent years, extreme global temperatures have been occurring more and more frequently due to anthropogenic and environmental changes. Sustained high temperatures can have a detrimental effect on plant growth and development. For example, extreme temperatures can reduce the viability of plant seeds, restrict germination and even limit plant survival (Yamori et al., 2014). As a result, it is critical to comprehend the plant’s heat tolerance. HSP is a highly conservative protein in organisms that is created when they are subjected to extreme temperatures (Gupta et al., 2010; Cui et al., 2021). Heat shock proteins are generally low in quantity under normal conditions, but when exposed to heat, they rapidly increase to account for up to 15% of total protein composition in organisms (Swindell et al., 2007; Huang et al., 2022).

Plant heat shock proteins (HSPs) respond to changes in the external environment by functioning as molecular chaperones, maintaining protein folding homeostasis, and preventing or correcting protein misfolding and degradation (Chang et al., 2006). HSP100, HSP90, HSP70, HSP60, and HSP20 are the five categories of plant HSPs based on their molecular weight and sequence homology (Waters, 2013). HSP20s are the most common and abundant proteins in plant HSPs, with molecular weights ranging from 15 to 42 kDa (Ji et al., 2019). Most HSP20s may form high-molecular-weight oligomers and are involved in protein stability. Hence, they play an essential role in the development of plant thermotolerance (Haslbeck and Vierling, 2015). For example, studies have found that heat stress will induce a large number of HSP20 genes in apple, pepper and pumpkin organisms (Guo et al., 2015; Yao et al., 2020; Hu et al., 2021). HSPs heat stress tolerance in transgenic organisms have also been confirmed by other research. For example, *PtHSP17.8* from HSP20 is a heat tolerance gene in *Populus trichocarpa* (Li et al., 2016). The rice HSP20 gene *OsHSP17.7* is involved in UV-B and heat stress (Murakami et al., 2004). Heat tolerance was improved in transgenic tobacco that overexpressed the *Zea mays* HSP20 gene *ZmHSP16.9* (Sun et al., 2012). HSP20 genes appear to be important in modulating heat tolerance in plants, according to these research.

The alpha-crystallin domain (ACD), which contains around 90 amino acid residues, is the most conspicuous characteristic of HSP20 protein (Waters et al., 1996). The HSP20 gene family, unlike some of the other HSPs, has a lot of sequence differences and evolutionary variability (Basha et al., 2012). There are 19–94 HSP20 genes frequently researched or commercially relevant plant species (Waters, 2013). Plant HSP20s can also be classified into diverse subfamilies based on their cellular sequence similarity, location, and functionality (Waters et al., 1996). HSP20 proteins in *Arabidopsis* were classified into 12 subfamilies (Waters, 2013), whereas soybean HSP20 proteins were grouped into 15 subfamilies (Lopes-Caitar et al., 2013), and *G. hirsutum* HSP20 proteins were divided into 14 subfamilies (Ma et al., 2016).

*Davidia involucrata* is a living fossil that is not only recognized as an “endangered” species in China, but also serves as a symbol for the surviving relict plants of ancient provenance (Manchester et al., 2009). Because of the co-occurrence of their natural ranges and the earliest specimens by the same explorer, *D. involucrata* has been renowned as an exceedingly old horticultural tree and is considered as the “giant panda” of plants. As a solanaceous plant, *D. involucrata* is highly resistant to abiotic stresses (Chen Y. et al., 2020). The molecular regulatory mechanism governing *D. involucrata* responses to abiotic stressors, on the other hand, is yet unknown. Global extreme temperature have been limiting dove tree growth and development in recent years. To increase the heat resistance of *D. involucrata*, it is critical to research the heat resistance mechanism of *D. involucrata* and select intense heat resistant variants. A study of the HSP20 gene family is valuable for understanding the heat tolerance mechanism of *D. involucrata.*

We evaluated the phylogenetic relationships, physicochemical characteristics, *cis*-elements, conserved domains, gene structures, and expression patterns of HSP20 genes identified using bioinformatics approaches based on the entire genome of *D. involucrata* (Chen Y. et al., 2020). This research lays the groundwork for future research into the roles of the *DiHSP20* gene family. Our findings may also provide light on the molecular significance of *DiHSP20* during heat stress.

## Materials and methods

### HSP20 genes in *D. involucrata*

The HSP20 domain’s Hidden Markov Model (HMM) profile (PF00011) were obtained from the Protein Family Database (Pfam 34.0; http://pfam.xfam.org/). *D. involucrata*’s whole genome, genome annotation, and protein sequence data were obtained from the National Genomics Data Center (https://bigd.big.ac.cn/?lang=en) under accession number PRJCA001721. HMMER research (E-value < 10^−5^) was used to find the candidate HSP20 protein sequences (Yu et al., 2016). The positions of the conserved HSP20 domain were validated using the Simple Modular Architecture Research Tool (SMART, v9; http://smart.embl.de/smart/batch.pl), and Conserved Domain Database (CDD, v3.19; https://www.ncbi.nlm.nih.gov/Structure/bwrpsb/bwrpsb.cgi). The ultimate potential *D. involucrata* HSP20 proteins were found after removing redundant sequences without a similar HSP20 domain or having a molecular weight beyond the range of 15–42 kDa (Ji et al., 2019). The candidate HSP20 proteins from *Oryza sativa* and *Arabidopsis thaliana* were collected using the same way, from the Rice Genome Annotation Project (http://rice.plantbiology.msu.edu/) and TAIR database (https://www.arabidopsis.org/index.jsp).

### Gene structure and sequence analysis

ExPASy’s pI/MW tool (v.3.0; http://web.expasy.org/protparam/) was used to calculate the theoretical isoelectric point (pI) and molecular weight (MW) of every HSP20 protein. The Gene Structure Display Server (GSDS 2.0; http://gsds.cbi.pku.edu.cn) was used to view the HSP20 gene structures. The MEME program (MEME Suite 5.3.3; http://meme-suite.org/tools/meme) has been used to find conserved motifs in HSP20 proteins, with the number of repeats (any, maximum number of motifs-10, and ideal motif widths set from 6 to 200 amino acid residues) being recorded. TBtools was used to visualize the findings (Chen C. et al., 2020).

### *Davidia involucrata* HSP20 gene phylogenetic analysis and classification

The CLUSTALW (Thompson et al., 1994) tool was used to align the complete amino acid sequences of 106 HSP20 members from *D. involucrata* (N = 42), *Arabidopsis* (N = 31), and *Oryza sativa* (N = 33). (Supplementary Table S1). MEGA-X (Kumar et al., 2018) had been used to create a phylogenetic tree by using Maximum Likelihood (ML) approach with a 1,000-fold bootstrap test, and the best matching model is the generalized time-reversible (GTR) model in conjunction with the Shimodaira–Hasegawa (SH) test. According to the categories and *in silico* subcellular localization of HSP20 proteins in *O. sativa* and *Arabidopsis*, the HSP20 proteins were divided into many categories (Waters et al., 1996). To use the EvolView web program (https://evolgenius.info/evolview-v2), the phylogenetic tree was displayed and enriched.

### Analysis of gene duplication and collinearity of *DiHSP20s*

The homologous sections of the *DiHSP20* genes were initially discovered using OrthoFinder software, and a synteny graph was created using TBtools based on the found homologous gene pairs (Chen C. et al., 2020). Multiple Collinearity Scan Toolkit (MCScanX) software was used to create syntenic maps (Wang et al., 2012) to show synteny links between orthologous HSP20 genes in *D. involucrata* and other focused species (*Arabidopsis*, *O. sativa* and *Camptotheca acuminata*). The size of the more minor gene covered was equivalent to or more than 70% of the larger gene, and the difference between the two focused genes was less than 30% to determine *DiHSP20* gene duplication occurrences. Tandem duplications were characterized by two or more contiguous duplication on the same chromosome in less than 100 kb, whereas segmental duplications were considered as duplicates on distinct chromosomes or at a distance more than 100 kb on the same chromosome (Kan et al., 2021).

### *DiHSP20* gene promoter analysis and 3D protein structure

According to the default settings, the tertiary structures of *DiHSP20* proteins were estimated utilizing online prediction program SWISS-MODEL (https://swissmodel.expasy.org/). The kinds, quantities, and functionality of *cis*-acting elements within that 1,500 bp upstream regions of the coding area of *D. involucrata* HSP20 genes were evaluated employing PlantCARE tools (http://bioinformatics.psb.ugent.be/webtools/plantcare/html/) obtained from the *D. involucrata* genome.

### Expression pattern of *DiHSP20* genes in different tissues and in response to heat stress

*Davidia involucrata* transcriptomic data was received from the NCBI public database (Accession number: PRJNA596897). *DiHSP20* genes expression was examined in various tissues, including the root, bract, leaf, and flower. The NCBI transcriptome database were also used to get the expression patterns of the *DiHSP20* genes that reacted to heat stress (Accession number: PRJNA524021). For analysis of the data, the transcription data was converted to log2 [transcripts per million (TPM)+1] values (Zeng et al., 2020). TBtools software was used to create a heatmap of the obtained gene expression patterns (Chen C. et al., 2020).

### Plant materials and heat stress treatment

Chengdu (102°54′E; 30°05′N) in Sichuan province, China, provided fresh and mature *D. involucrata* propagules. The propagules were grown in plastic pots with sand in a growth chamber (25°C temperature, 75% humidity, 14 h light/10 h darkness photoperiod) and irrigated weekly with 1/2-strength Hoagland’s nutritional solution. The seedlings were developed under heat stress treatment (42°C) for 0, 1, 6, and 12 h at the six-leaf stage. Three seedlings were included in each treatment. The leaves were sampled and instantly frozen in liquid nitrogen.

### qRT-PCR validation of *DiHSP20* gene expression

RNASimple Total RNA Kit (Tiangen, Beijing, China) was used to extract total RNA, and TIANScript cDNA kit (Tiangen, Beijing, China) was used to generate cDNA according to the manufacturer’s instructions. Following total RNA extraction and cDNA synthesis, quantitative analysis was carried out using a CFX96 Touch Real-Time PCR Detection System (Bio–Rad, United States) and SuperReal PreMix Plus (SYBR Green, Tiangen, China) according to the manufacturer’s instructions. The PCR program included an initial denaturation step at 95°C for 30 s, followed by 40 cycles of 95°C for 10 s, 58°C for 10 s, and 72°C for 30 s. The 2^−ΔΔCT^ approach was used to determine the relative expression level of the focused genes (Livak and Schmittgen, 2001). All data were calculated using the expression level under heat stress divided by that under normal condition at the same time points and presented as the means ± standard error (SE) of three replicates and differences were detected using the Student’s t-test. Asterisk (* or **) indicate a significant difference at *p* < 0.05 or 0.01, respectively. Supplementary Table S2 lists all of the primers utilized.

## Results

### HSP20 proteins in *D. involucrata*

A HMM search of the *D. involucrata* genome database yielded seventy-three HSP20 proteins. After eliminating duplicated and insufficient sequences, as well as those with something like a molecular weight outside the 15–42 kDa region, 42 sequences were identified as HSP20 genes and given names based on their chromosomal positions. It content information on each *DiHSP20* gene, including its name, identity, chromosomal position, amino acid (AA) number, genomic location, isoelectric point (pI), and molecular weight (MW) (Table 1). *DiHSP20* genes have been identified on 15 chromosomes of *D. involucrata*. The *DiHSP20* proteins had an average of 229 AAs and varied in size from 136 (*DiHSP20-38*) to 643 (*DiHSP20-27*). *DiHSP20s* had MWs ranging from 15.65 kDa (*DiHSP20-38*) to 42 kDa (*DiHSP20-27*), with an average of 25.03 kDa, and pI values of 4.76 (*DiHSP20-23*) to 9.83 (*DiHSP20-25*), with an average of 6.65.

**TABLE 1:** Features of *DiHSP20* genes in *D. involucrata*.

Gene name	Gene ID	Chr	Genomic location	AA	MW(Kda)	pI	Type
*DiHSP20-1*	GWHPABJS070359	Chr0	74343–74777	294	33.49	5.60	CI
*DiHSP20-2*	GWHPABJS070505	Chr0	1580638–1581087	294	33.48	5.60	CI
*DiHSP20-3*	GWHPABJS069566	Chr7	2933690–2933700	156	17.86	5.59	CI
*DiHSP20-4*	GWHPABJS069924	Chr7	6139200–6139659	156	17.85	5.60	CI
*DiHSP20-5*	GWHPABJS071778	Chr7	16536–16546	156	17.85	5.59	CI
*DiHSP20-6*	GWHPABJS029566	Chr17	32724107–32724117	156	17.76	5.40	CI
*DiHSP20-7*	GWHPABJS000537	Chr0	27813936–27814400	154	17.84	5.56	CI
*DiHSP20-8*	GWHPABJS026449	Chr16	6097182–6097207	161	18.22	7.91	CI
*DiHSP20-9*	GWHPABJS026455	Chr16	6158964–6159381	341	37.90	5.13	CI
*DiHSP20-10*	GWHPABJS000536	Chr0	27802532–27802610	147	17.06	6.22	CI
*DiHSP20-11*	GWHPABJS021884	Chr14	31522727–31522805	147	17.06	6.22	CI
*DiHSP20-12*	GWHPABJS040956	Chr20	4562935–4563265	435	41.90	6.16	CV
*DiHSP20-13*	GWHPABJS040964	Chr20	4590958–4591209	232	25.99	6.23	CV
*DiHSP20-14*	GWHPABJS070363	Chr2	128139–128687	332	37.45	4.90	CI
*DiHSP20-15*	GWHPABJS070501	Chr2	1526707–1527156	332	37.45	4.90	CI
*DiHSP20-16*	GWHPABJS070360	Chr2	90537–90561	164	18.63	5.54	CI
*DiHSP20-17*	GWHPABJS070504	Chr6	1568561–1569030	164	18.63	5.54	CI
*DiHSP20-18*	GWHPABJS070362	Chr6	115779–115970	213	24.25	6.22	CI
*DiHSP20-19*	GWHPABJS070502	Chr7	1541788–1542237	213	24.25	6.22	CI
*DiHSP20-20*	GWHPABJS060438	Chr7	45824686–45825092	231	26.30	8.47	P
*DiHSP20-21*	GWHPABJS026453	Chr16	6145374–6145433	170	19.35	5.24	CI
*DiHSP20-22*	GWHPABJS012176	Chr11	1957854–1958284	230	25.59	6.65	P
*DiHSP20-23*	GWHPABJS070361	Chr11	96483–96764	205	23.21	4.76	CI
*DiHSP20-24*	GWHPABJS070503	Chr11	1549191–1549251	200	22.67	4.89	CI
*DiHSP20-25*	GWHPABJS013327	Chr11	11421667–11422269	317	36.64	9.83	CI
*DiHSP20-26*	GWHPABJS006383	Chr1	41537159–41537375	240	27.10	8.87	CV
*DiHSP20-27*	GWHPABJS067170	Chr9	46203574–46203696	643	42.00	9.41	CI
*DiHSP20-28*	GWHPABJS035031	Chr19	31803710–31803842	140	15.66	6.74	CIV
*DiHSP20-29*	GWHPABJS056172	Chr6	2341462–2341714	238	26.68	8.87	P
*DiHSP20-30*	GWHPABJS019946	Chr13	44700683–44700914	278	31.04	5.6	CV
*DiHSP20-31*	GWHPABJS012771	Chr11	5879469–5879866	301	33.45	9.49	MII
*DiHSP20-32*	GWHPABJS012772	Chr11	5879469–5879887	308	34.23	9.25	MII
*DiHSP20-33*	GWHPABJS053971	Chr5	5328148–5328512	190	21.34	5.40	MII
*DiHSP20-34*	GWHPABJS053975	Chr5	5352487–5352905	213	24.18	5.43	MII
*DiHSP20-35*	GWHPABJS053972	Chr5	5334809–5335176	193	21.37	5.54	MII
*DiHSP20-36*	GWHPABJS070364	Chr9	140163–140188	137	15.89	8.09	CI
*DiHSP20-37*	GWHPABJS070500	Chr9	1518974–1519361	137	15.89	8.09	CI
*DiHSP20-38*	GWHPABJS051796	Chr4	45229588–45229856	136	15.65	6.08	ER
*DiHSP20-39*	GWHPABJS009975	Chr10	7455479–7455876	187	21.32	9.25	MII
*DiHSP20-40*	GWHPABJS039961	Chr2	32356776–32356841	213	23.89	8.99	ER
*DiHSP20-41*	GWHPABJS039959	Chr2	32353746–32353748	270	30.47	9.44	ER
*DiHSP20-42*	GWHPABJS001872	Chr0	42480645–42480951	198	22.54	4.94	MI

### *DiHSP20* proteins phylogenetic analysis

**FIGURE 1:**
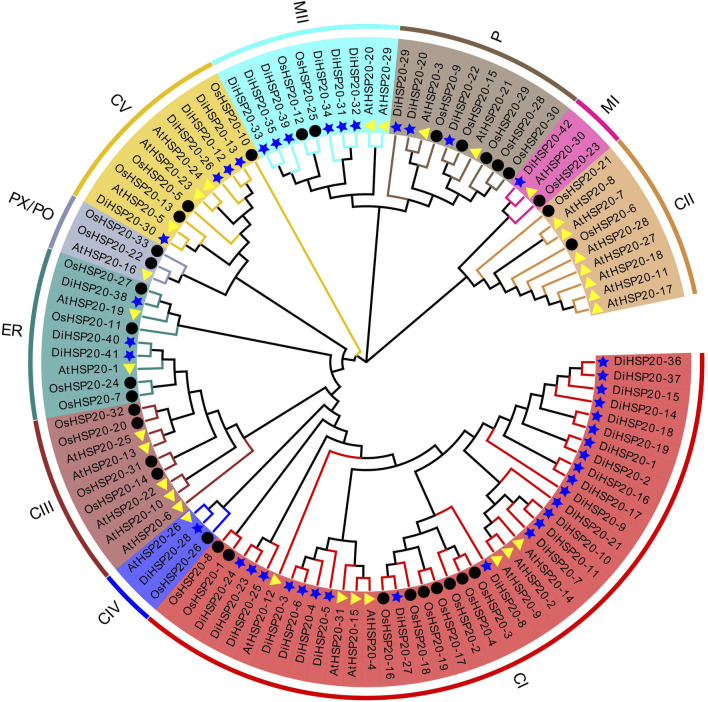
Phylogenetic analysis of *Davidia involucrata*, *Arabidopsis*, and rice HSP20s proteins. MEGA-X software was used to create a phylogenetic tree of HSP20 proteins. The ten subgroups are denoted by various colors. *D. involucrata* HSP20s (*DiHSP20*s), *Arabidopsis* HSP20s (*AtHSP20*s), and rice HSP20s (*OsHSP20*s) are shown by blue stars, yellow triangles, and black circles, respectively.

A ML phylogenetic tree was generated to analyze the evolutionary connections of *D. involucrata* HSP20 proteins. HSP20 proteins were classified into seven subfamilies: cytosol I (CI), CIV, CV, mitochondria I (MI), MII, endoplasmic reticulum (ER), and plastid (P). The cytoplasmic subfamilies (CI through CV) were the biggest clade, with 29 members, whereas subfamilies CII and CIII had no *DiHSP20s*. Seven gene members were detected in the M (MI and MII) subfamilies, three in the ER subfamilies, and three in the P subfamilies, whereas no *DiHSP20s* were found in the PX/Po subfamily (Figure 1; Table 1).

### *DiHSP20s* gene structures and conserved motifs

**FIGURE 2:**
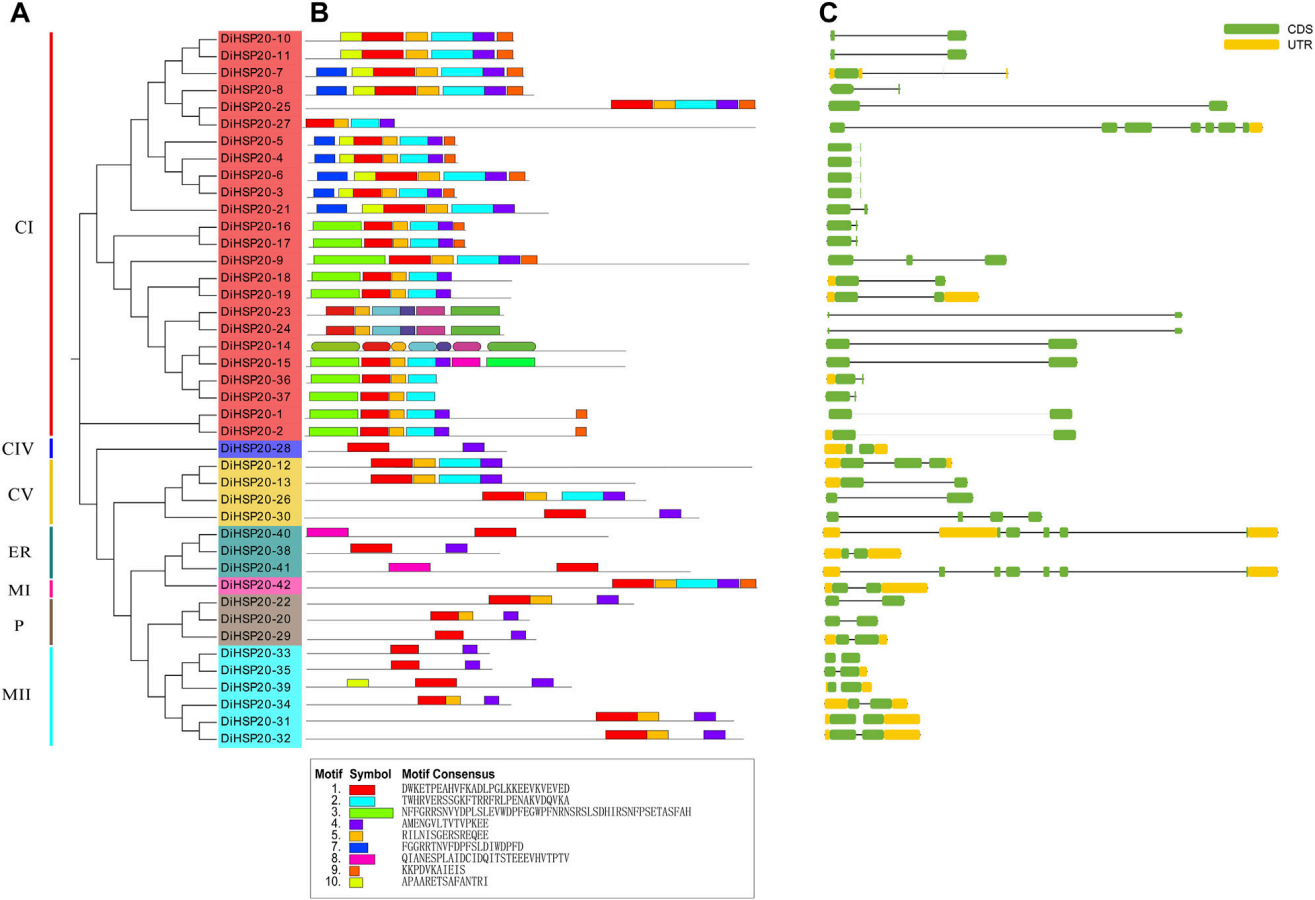
Members of the *DiHSP20* family’s phylogenetic connections, structures, and motifs **(A)** Maximum Likelihood techniques were used to generate a phylogenetic tree comprising 42 *DiHSP20* proteins. Different backdrop colors and letters are used to denote the different groupings **(B)** *DiHSP20* proteins’ conserved motifs. Different motifs are represented by colored boxes and different numbers **(C)** The *DiHSP20* genes’ exon/intron architecture. Green boxes, black lines, and yellow boxes correspondingly indicate exons, introns, and UTRs. With the help of TBtools, we were able to estimate the phylogenetic tree, conserved motifs, and gene structures.

The conserved motifs were examined by MEME to study the structural properties of the HSP20 proteins. A total of ten different motifs, numbered 1 through 10, were identified. These preserved motifs ranged in length from 6 AAs (motif 9) to 50 AAs (motif 6). The amount of conserved motifs varied from 2 to 7 for every HSP20 protein, while most *DiHSP20s* had 6 conserved motifs (Figure 2B).

The exon-intron architecture of *D. involucrata* HSP20 genes were studied to reveal their evolutionary relationships (Figure 2C). 23 (55 percent) of the HSP20s had no introns, 11 (26 percent) had two introns, 6 (14 percent) had one intron, and two genes (*DiHSP20-7* and *DiHSP20-40*) had three introns. Genes with comparable exon-intron patterns were clustered together using gene structure analysis (Figures 2A,C).

### The *DiHSP20* proteins’ homology modeling

**FIGURE 3:**
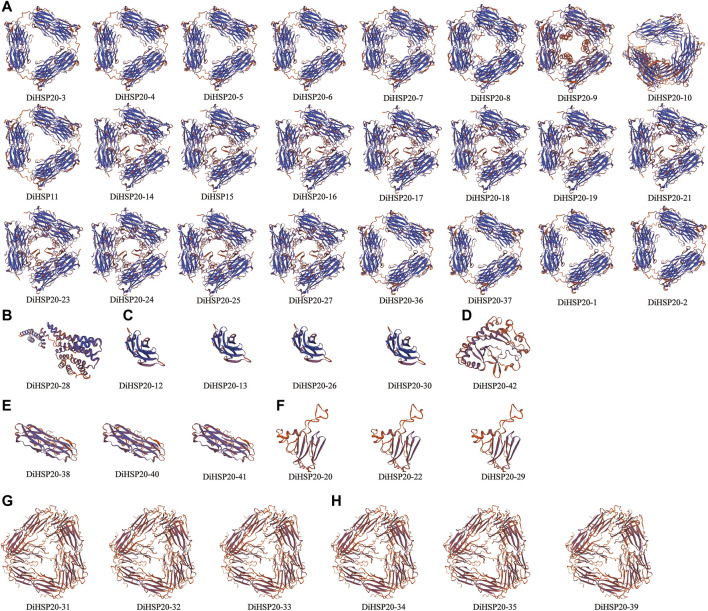
The anticipated 3-dimensional structural models of *DiHSP20* proteins are depicted in cartoon form. The distinct categories are represented by capital letters **(A–H)**.

Protein homology modeling was done utilizing a SWISS-MODEL platform to determine a suitable theoretical structure of the *DiHSP20s*. Figure 3 shows the expected 3D structures. Every *DiHSP20* protein was programmatically searched for a template in the program, and then a model was generated utilizing the template. According to their structural similarities, all proteins may be grouped into seven categories (groups A through G). The most proteins were found in Group A, which had 24. Only one protein was found in groups B (*DiHSP20-28*). Group C had 4 proteins. Group D and F had three proteins. Every *DiHSP20* protein contained *β*-turn, and the protein structures of the same template were almost identical, implying that the expected findings were accurate.

### *DiHSP20s* gene duplication, chromosomal location and synteny analysis

**FIGURE 4:**
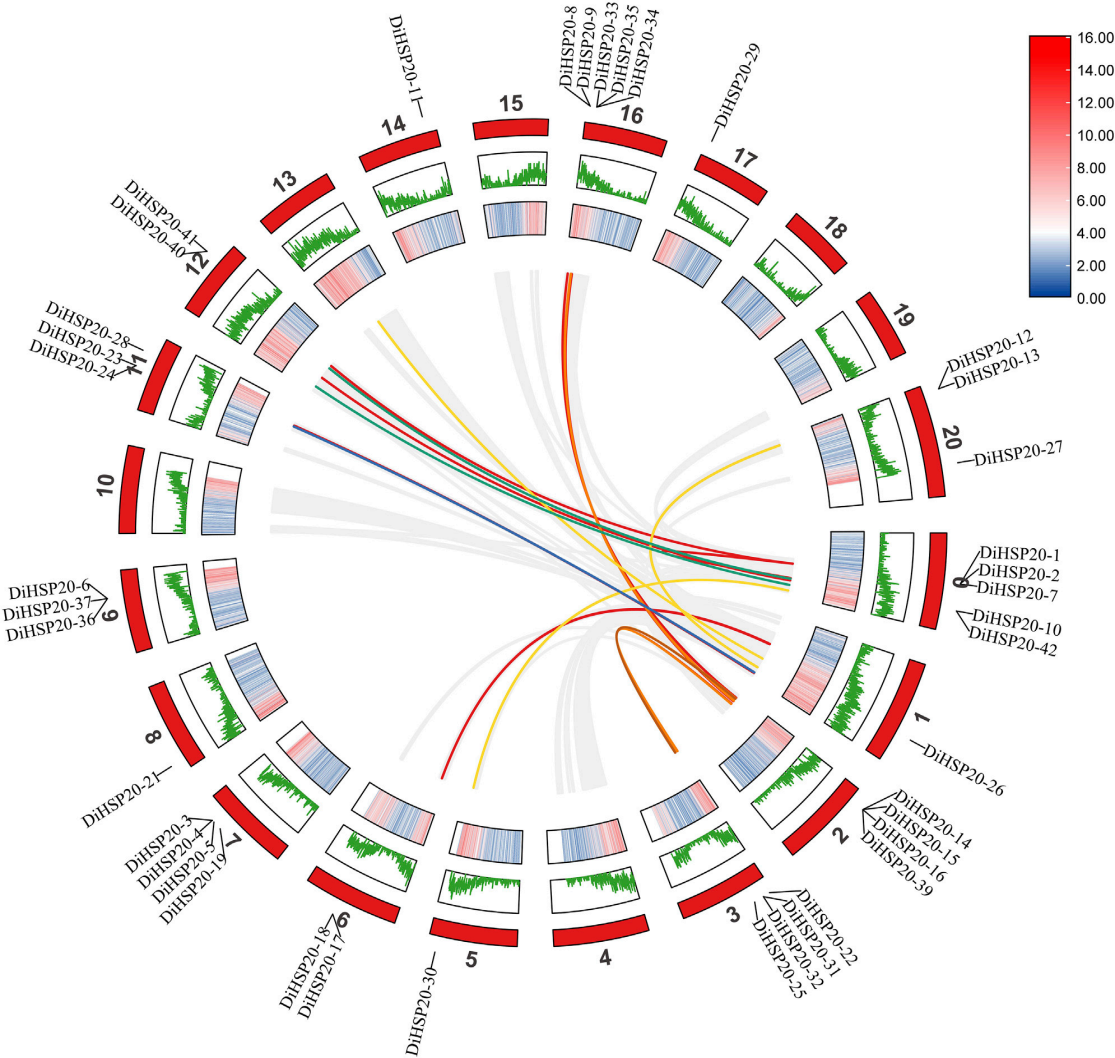
Synteny analysis between HSP20 genes of *D. involucrata*, as performed by MCScanX. The chromosome numbers of *D. involucrata* are 0–20. The outermost circle represents the length of the chromosome, the second circle represents the GC content and the third circle represents gene density. The curves indicate synteny between HSP20 genes within *D. involucrata.*

A total of 42 *DiHSP20* genes were identified and distributed unevenly throughout 15 chromosomes (Chr) (Figure 4). The largest number of *DiHSP20* genes per Chr was five (Chr00 and Chr16), whereas Chrs 00, 05, 08, 14, and 17 had just one gene. The investigation of gene duplication events revealed that the *D. involucrata* HSP20 gene family had two pairs of tandem duplication genes (*DiHSP20-1/-2* and *DiHSP20-40/-41*) and two pairs of segmentally duplicated genes (*DiHSP20-8/-14* and *DiHSP20-24/-26*) according to the stated criteria (Figure 4). The sequence similarity was 74.55%, 80.11%, 78.66% and 82.11%, respectively.

Orthofinder was used to discover orthologous genes and TBtools was used to create a comparative synteny map to further investigate the synteny links of *DiHSP20* genes within *D. involucrata* (Figure 4). 30 orthologous HSP20 gene pairs have been identified based on these genes and syntenic loci in *D. involucrata* chromosomes, implying that the majority of HSP20 genes in *D. involucrata* have a similar origin and evolutionary process.

**FIGURE 5:**
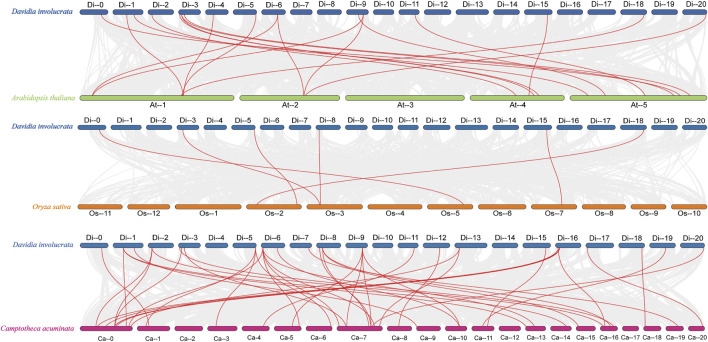
Synteny analyses of HSP20 genes between *D. involucrata* and three other representative plant species (*Arabidopsis*, rice, *C. acuminata*) **(A)** *D. involucrata* VS. *A. thaliana*. **(B)** *D. involucrata* VS. *O. sativa*. **(C)** *D. involucrata* VS. *C. acuminata*. Gray lines indicate significantly collinear blocks within and among plant genomes, while red lines highlight syntenic HSP20 gene pairs. The chromosome number is indicated at the top of each chromosome.

Comparative synteny maps of three related genomes (*D. involucrata* VS. *A. thaliana*, *D. involucrata* VS. *O. sativa* and *D. involucrata* VS. *C. acuminata*) were generated to better understand the evolutionary connections of the HSP20 genes with several other species. Nineteen *DiHSP20* genes of *D. involucrata* shared a syntenic connection with *Arabidopsis* genes, 39 with *C. acuminata* genes, and just six with *O. sativa* genes (Figure 5). The amount of collinear gene pairings between *D. involucrata* and other Nyssaceae members (*C. acuminata*) was higher than that between more distantly related *Arabidopsis* or *O. sativa*, with *D. involucrata* and *O. sativa* having the fewest.

### Promoter analysis of *DiHSP20* genes

**FIGURE 6:**
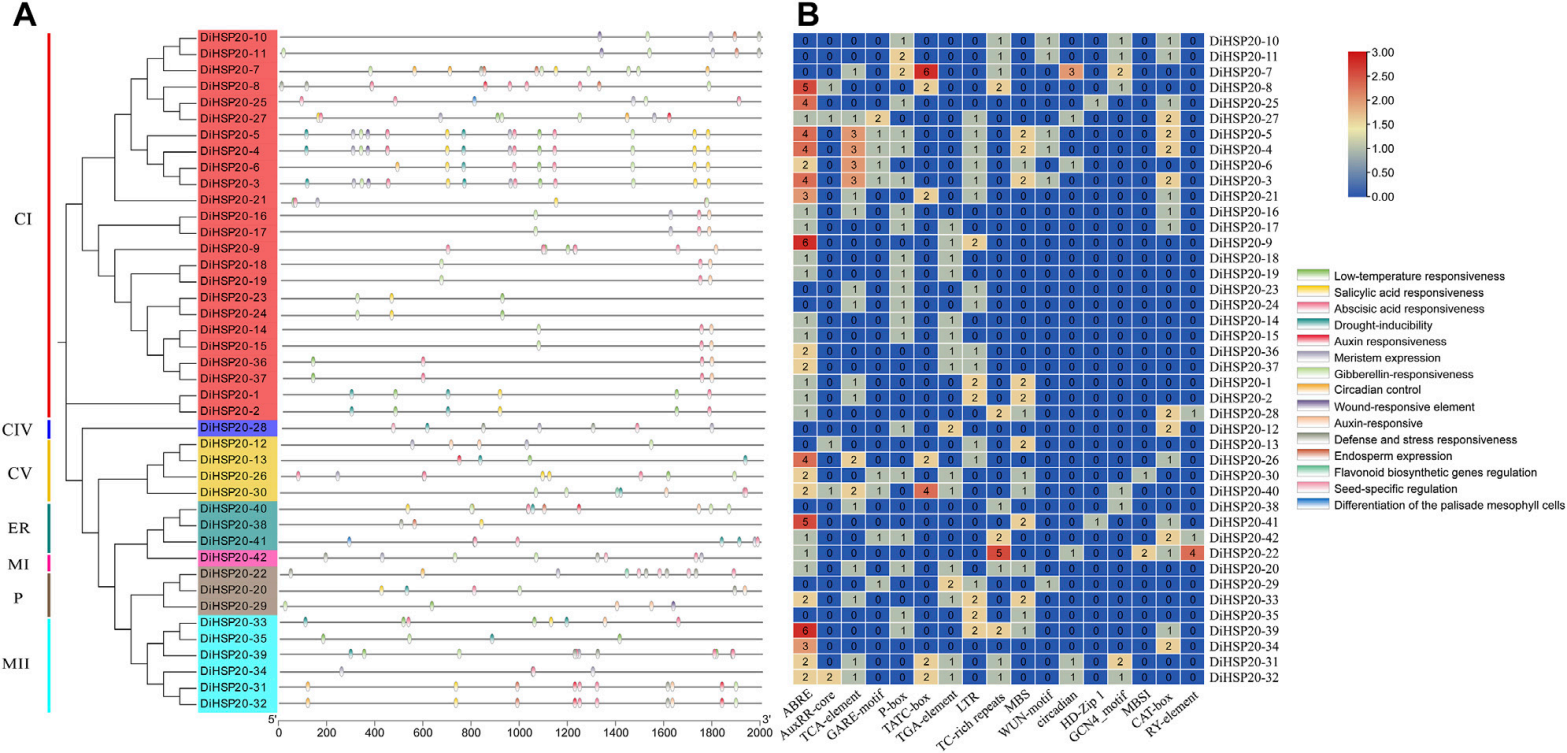
Cis-elements evaluation of the *DiHSP20* gene promoter regions **(A)** The distinct colors and numbers in the *DiHSP20* genes showed the variety of different promoter elements. **(B)** Different kinds of cis-elements and relative positions in every *DiHSP20* gene are represented by colored blocks.

To further understand the mechanism of *cis*-regulatory elements in *DiHSP20*, *cis*-elements were detected in the 1.5 kb upstream of every *DiHSP20*’s translation start site (ATG) (Figure 6). Three categories of *cis*-elements, including stress responsive, plant development-related and hormone responsive elements have been discovered. The most *cis*-elements found among the 31 *DiHSP20* genes were connected with hormone responsiveness, such as ABRE. Auxin responsiveness (AuxRR-core and TGA-element), abscisic acid responsiveness (ABRE), gibberellin responsive elements (GARE-motif, P-box, and TATC-box), and salicylic acid-responsive (TCAelement) were all found in abundance in the promoter region. The hormone responsive elements ABRE, TCAelement, and TGA-element made up the majority of the elements, whereas the AuxRR-core element was only discovered in the promoter region of five *DiHSP20* genes. The category for stress-related issues abiotic stress-related elements (LTR, TC-rich repeats, and MBS) and biotic stress-related elements (LTR, TC-rich repeats, and MBS) are found in *cis*-elements (WUN-motif). Circadian control (circadian), palisade mesophyll cell differentiation (HD-Zip 1), endosperm expression (GCN 4 motif), meristem expression (CAT-box), flavonoid biosynthesis genes regulation (MBSI), and seed-specific regulation (RYelement) were all identified as plant development-related elements. Only the promoter regions of *DiHSP20-25* and *DiHSP20-41* included the HD-Zip 1 element, whereas the promoter regions of *DiHSP20-28*, *DiHSP20-42*, and *DiHSP20-22* contained the RY-element element (Figure 6).

### *DiHSP20* gene expression patterns in different organs and in response to heat stress

**FIGURE 7:**
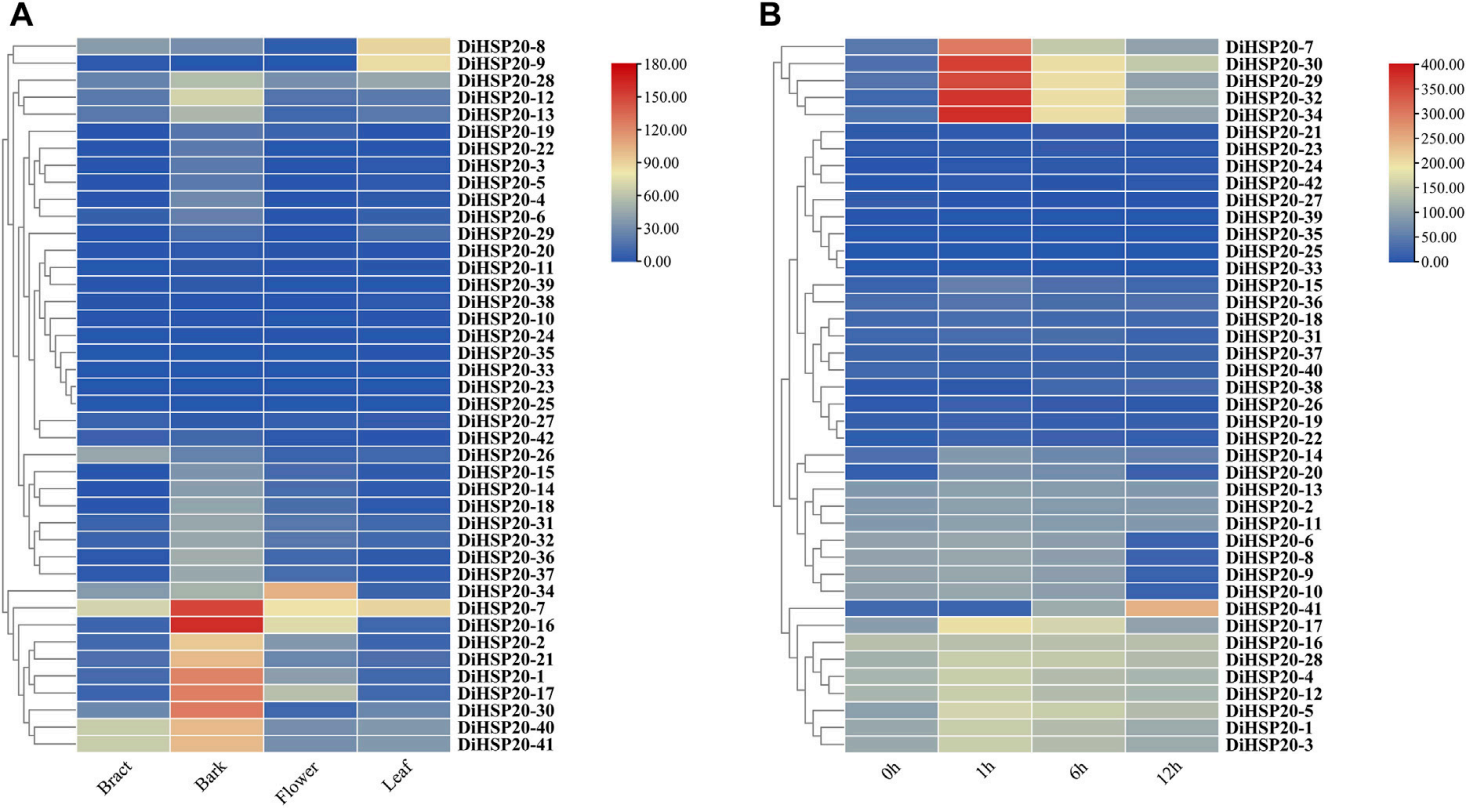
*DiHSP20* gene expression in *D. involucrata* **(A)** Based on publically accessible transcriptome data, the transcript levels of the *DiHSP20* genes in four tissues of *D. involucrata* were studied. From blue to red, the color scale reflects increasing degrees of expressiveness. **(B)** Based on publically accessible transcriptome data, the transcript levels of *DiHSP20* genes in response to heat stress were studied.

The expression patterns of all 42 *DiHSP20* genes were studied using a conventional transcriptome analysis approach based on publically accessible transcriptomic data from *D. involucrata* tissues such as bark, bracts, leaves, and flowers. Among the 42 *DiHSP20s*, 39 were expressed in tissues above (TPM >0). A few really *DiHSP20* genes were expressed preferentially in all of the tissues studied. *DiHSP20* genes were most significantly expressed in the bark, particularly *DiHSP20-7*, *DiHSP20-16*, *DiHSP20-1*, *DiHSP20-17*, and *DiHSP20-30*. The most highly expressed *DiHSP20* genes in bract were *DiHSP20-7*, *DiHSP20-40* and *DiHSP20-41*. The most highly expressed *DiHSP20-34*, *DiHSP20-7*, *DiHSP20-16* and *DiHSP20-17* in Flower. The most highly expressed *DiHSP20* in leaf were *DiHSP20-8*, *DiHSP20-9* and *DiHSP20-7*. *DiHSP20-23*, *DiHSP20-25*, and *DiHSP20-33*, on the other hand, were minimally expressed in any of the tissues examined (Figure 7A).

The expression of *DiHSP20*s following heat treatment was investigated using publically accessible transcriptome data to study the potential roles of *DiHSP20*s in response to heat stress. The results showed that the expression levels of most *DiHSP20* genes, especially *DiHSP20-7*, *DiHSP20-29*, *DiHSP20-30*, *DiHSP20-32* and *DiHSP20-34* were sharply upregulated after 1 hour of heat treatment, the expression of which increased more than two-fold. The expression of most *DiHSP20* genes was heavily up-regulated at 1 h after heat stress, while it started to be down-regulated at 6 h and continued to decrease after 12 h. However, there were also some genes whose expression levels did not change after heat stress (Figure 7B).

**FIGURE 8:**
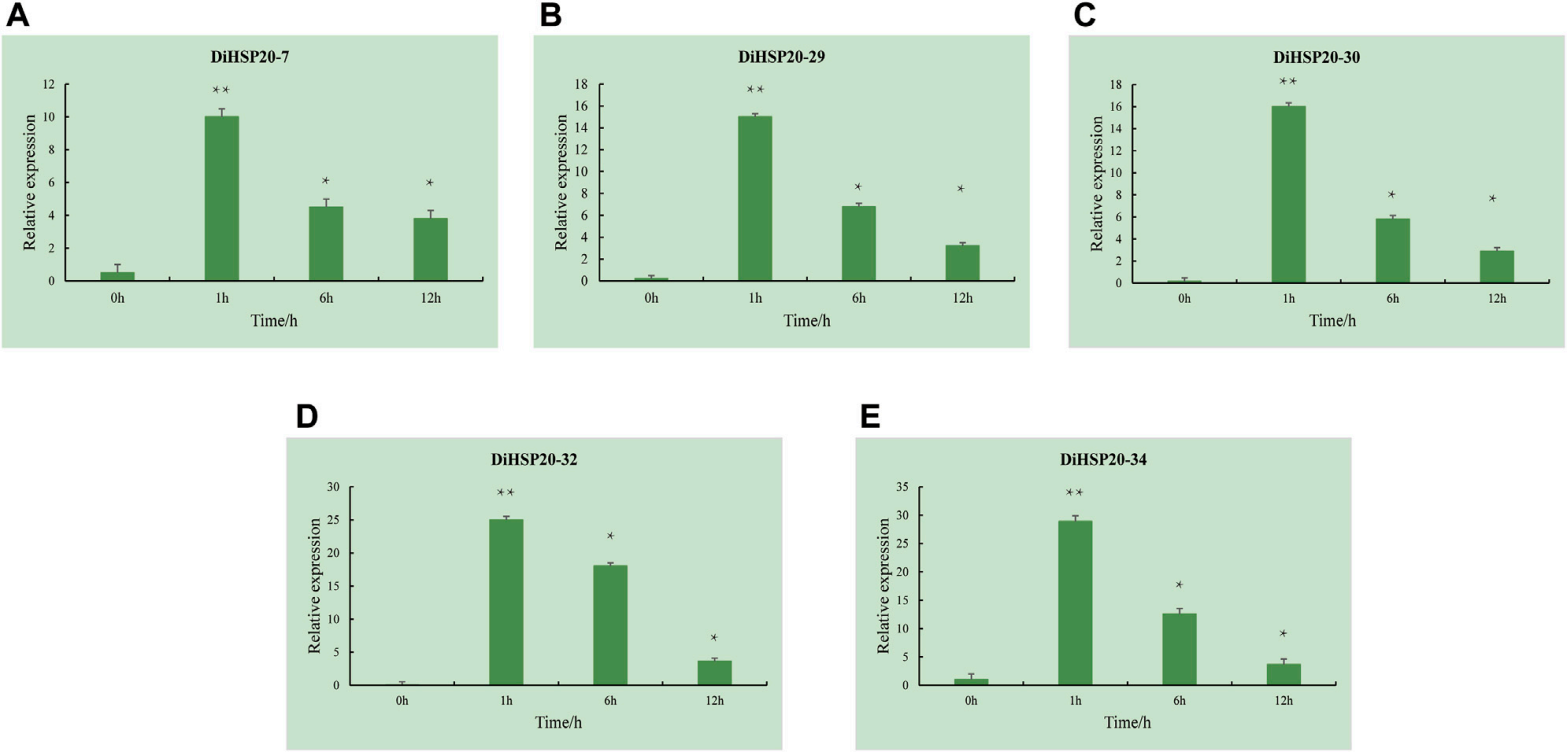
Expression profiles of 5 selected *DiHSP20* genes in response to heat stress. Asterisk (p or pp) indicate a significant difference at *p* < 0.05 or 0.01, respectively.

Five DEGs (*DiHSP20-7*, *DiHSP20-29*, *DiHSP20-30*, *DiHSP20-32*, and *DiHSP20-34*) were selected for expression analysis of the candidate *DiHSP20* genes that are critical for heat tolerance, and their expression levels were evaluated by qRT–PCR. Under 42°C, the expression of all of the investigated *DiHSP20* genes was extremely upregulated, as shown in Figure 8. At 1 hour, the expression of all candidate genes reached a peak and the level was much higher than the control group’s. After six and 12 h, the expression decreased sequentially, but the expression at this time was also much higher than that before the heat stress.

## Discussion

Climate variability is one of the world’s most pressing environmental and economic problems, with the potential to significantly reduce forest output and quality throughout forestal systems. Plant heat-shock proteins have been extensively found in plants and are rapidly produced in response to stress (Wang et al., 2004). Numerous studies have demonstrated the members of the HSP20 transcription factor generate heat shock proteins and play an important role in plant abiotic stress (Yu et al., 2016; Zhao et al., 2018). The roles of the HSP20 family proteins in numerous species have already been identified, such as the model species rice (Sarkar et al., 2009) and *Arabidopsis* (Scharf et al., 2001), along with soybean (Lopes-Caitar et al., 2013) and potato (Zhao et al., 2018). A detailed identification and characterization of the HSP20 gene in *D. involucrata* would offer essential information to this accumulated understanding while exposing their roles in *D. involucrata* stress tolerance.

It is now possible to evaluate members of the HSP20 gene family at the complete genome level thanks to the effective and efficient genome sequencing of *D. involucrata* (Chen Y. et al., 2020). Bioinformatics approaches were used to identify 42 HSP20 gene family members from the *D. involucrata* genome database. We discovered that the sample size of HSP20 genes in *D. involucrata* was not much different from *Arabidopsis* (N = 31) or rice (N = 33), but less than *Gossypium hirsutum* (N = 94) (Ma et al., 2016). These data point to the likelihood of a gene increase occurrence throughout the process of evolution from diploid (*Arabidopsis*, rice, *D. involucrata*) to tetraploid (*G. hirsutum*).

We built a phylogenetic relationships depending on the AA sequence data of *D. involucrata*, *Arabidopsis* and rice HSP20s to identify the evolutionary connections of HSP20 genes. Our phylogenetic study revealed that the HSP20 family in *D. involucrata* may be split into seven subfamilies, with 29 HSP20 genes in the cytoplasm belonging to CI, CIV, and CV, 7 genes in mitochondria belonging to MI and MII, 3 in the endoplasmic reticulum, and 3 in the plastids. These clustering patterns are almost similar to the distribution features of HSP20 family members in *Arabidopsis* (Scharf et al., 2001) and rice (Sarkar et al., 2009), showing a tight link between HSP20s from *D. involucrata*, *Arabidopsis*, and rice. This means that depending on the function of homologous genes in other species, the biological role of *D. involucrata* HSP20s might be inferred. Furthermore, over half of *DiHSP20*s were categorized into the CI-CV subfamilies, indicating that the cytoplasm may be the HSP20 family’s principal functional region in *D. involucrata*. Interestingly, none of the HSP20 genes belonged to the CII and CIII family, which contradicted the findings from the other focused species (Lopes-Caitar et al., 2013; Guo et al., 2015; Yao et al., 2020). The CII and CIII subfamilies may have appeared prior to *D. involucrata* speciation as a result of multiple gene duplication. Surprisingly, *DiHSP20* members from *D. involucrata* were much more strongly related to members of the same subfamily from different species than to other HSP20s from the same species, indicating high level of synteny across members of the same HSP20 subfamily from various plant species.

Exon/intron structure has played a key role in the development of multiple gene families (Xu et al., 2012). Approximately 79 percent of *DiHSP20* genes (mostly from the CI and CV subfamilies) contain only one intron or have no intron at all (Figure 2). This is not surprising, given that plants prefer to keep genes with no or few introns (Mattick and Gagen, 2001). Under environmental stress, the HSP20 gene family is one of the most quickly expressed genes (Sarkar et al., 2009; Yu et al., 2016). We investigated the expression levels of *D. involucrata* HSP20 genes in response to heat stress and discovered that the majority of HSP20 genes were significantly up-regulated.

The presence of a homologous domain was also explored in order to learn more about the development of HSP20 genes in *D. involucrata*. The results show that 6 conserved motifs were found in the majority of *DiHSP20*s, and motif 1 was found in approximately all of the proteins. Moreover, we discovered that most HSP20 genes from the same subfamilies have conserved motifs and similar gene architectures, which was similarly discovered in tomato and apple (Yu et al., 2016; Yao et al., 2020). This occurrence validated the subfamilies’ close evolutionary connection and categorization. These finding of the *DiHSP20* family’s conserved motifs and gene architectures may aid in the discovery of other activities of *DiHSP20* genes in *D. involucrata*, including in responses to various types of stressors.

Gene duplication phenomena are important in genomic reconfigurations and contractions (Vision et al., 2000), making plants possible to adapt to changing climatic conditions by increasing functional divergences (Flagel and Wendel, 2009). The 42 *DiHSP20* genes were irregularly distributed on *D. involucrata*’s 15 chromosomes, with 10 clusters containing at least two *DiHSP20* genes apiece (Figure 4). In several parts, particularly in Chrs 00 and 16, a group of family members congregated into clusters. Two sets of projected tandem duplicated HSP20 genes and two pairs of expected segmental duplicated HSP20 genes were detected, implying that the two duplication events had contributed to the expansion of the *DiHSP20* family.

Furthermore, the promoter regions of *DiHSP20* genes contained multiple hormone-responsive, stress-responsive, and plant development-related components (Figure 6), revealing that the HSP20 genes in *D. involucrata* have several or distinct roles. HSP20s have also been shown to play a critical part in the regulation of plants’ responses to environmental stress (Sarkar et al., 2009; Yu et al., 2016). The majority of the 42 *DiHSP20* genes were found to be up-regulated in response to heat stress, after 1 hour of heat treatment, the expression of *DiHSP20-7*, *DiHSP20-29*, *DiHSP20-30*, *DiHSP20-32*, and *DiHSP20-34* was markedly increased (Figure 7B). The expression levels of five DEGs (*DiHSP20-7*, *DiHSP20-29*, *DiHSP20-30*, *DiHSP20-32*, and *DiHSP20-34*) were assessed using qRT–PCR to corroborate the potential *DiHSP20* genes that are crucial for heat tolerance. It is worth mentioning that after 1 h of heat stress, the relative expression levels of the 5 HSP20 genes were substantially up-regulated. These genes may play a role in the heat stress biological pathway and could be exploited as candidate genes for *D. involucrata* heat resistance breeding.

## Conclusion

A genome-wide analysis of HSP20 proteins in *D. involucrata* was undertaken in this work, and 42 *DiHSP20* genes were found. According to their evolutionary links, the 42 *DiHSP20* genes are unequally distributed across 16 chromosomes and were grouped into seven subfamilies. The fundamental properties, genome location, gene architectures, homologous motifs, gene duplication phenomena, and *cis*-elements of the 42 genes were studied, to provide a critical understanding of the HSP20 gene family’s evolutionary relationships. The expression of *DiHSP20* genes was investigated using qRT-PCR, and the findings indicated that heat stress up-regulated the expression of 5 candidate *D. involucrata* HSP20 genes. Our findings offer a foundation for identifying key potential HSP20 genes implicated in *D. involucrata* heat stress responses (Yang et al., 2008).

## Data Availability

The datasets presented in this study can be found in online repositories. The names of the repository/repositories and accession number(s) can be found in the article/Supplementary Material.
